# Reptile Exposure in Human Salmonellosis Cases and *Salmonella* Serotypes Isolated from Reptiles, Ontario, Canada, 2015–2022

**DOI:** 10.3201/eid3110.241803

**Published:** 2025-10

**Authors:** Katherine Paphitis, Alexandra Reid, Hannah R. Golightly, Janica A. Adams, Antoine Corbeil, Anna Majury, Allana Murphy, Heather McClinchey

**Affiliations:** Public Health Ontario, Toronto, Ontario, Canada (K. Paphitis, J.A. Adams, A. Corbeil, A. Majury, A. Murphy); Ontario Ministry of Agriculture, Food and Agribusiness, Guelph, Ontario, Canada (A. Reid, H.R. Golightly); Ontario Ministry of Health, Toronto (H. McClinchey)

**Keywords:** salmonellosis, bacteria, animal salmonella infections, disease outbreaks, public health surveillance, reptiles, salmonella, zoonoses, Canada

## Abstract

Reptile-associated outbreaks of human *Salmonella* infections are increasing in Canada, coinciding with a rise in the popularity of reptiles as pets. We conducted a retrospective analysis of surveillance data for human *Salmonella* case-patients in Ontario during 2015–2022. We compared serotypes and reptile types for those reporting domestic reptile or amphibian exposure with veterinary *Salmonella* isolates reported during the same period. Case-patients commonly reported contact with reptile types from which *Salmonella* was most frequently isolated. Some serotypes from human case-patients were closely associated with contact with specific reptile types, including *Salmonella* Paratyphi B biovar Java (*Salmonella* Paratyphi B variant L (+) tartrate +) with snakes, *Salmonella* Agbeni with turtles, and *Salmonella* Cotham, *Salmonella* Chester, and *Salmonella* Tennessee with bearded dragons. *Salmonella* was most likely to be reported from reptiles fed a carnivorous diet. Education of reptile owners could help promote proper veterinary care and reduce transmission of zoonotic infections.

*Salmonella* is a common cause of human bacterial enteric illness in Canada (*1*). Most cases of human infection are associated with *Salmonella enterica* subspecies *enterica*, the main subspecies responsible for mammalian salmonellosis and one that is also frequently isolated from reptiles (*2*,*3*). Each year, a lesser number of human infections are associated with *Salmonella* subspecies primarily associated with cold-blooded animals, including reptiles, such as *S*. *enterica* subspecies *salamae*, *arizonae*, *diarizonae*, *houtenae*, and *indica* (*2*). *Salmonella* bacteria are naturally found in the gastrointestinal microbiotia of many animals, including reptiles kept as pets. According to a representative national population-based survey in Canada (2014–2015) assessing food, animal and water exposures, 2.1% of Ontario residents reported contact with a reptile in the week before interview, and 1.0% reported contact with an amphibian (*4*). A recent review by Varela et al. (*5*) noted ownership of pet reptiles and amphibians is increasing in the United States; similar trends have been reported in Canada, particularly during the COVID-19 pandemic when persons were spending more time at home (*6*).

Reptiles (including lizards, snakes, and turtles) and amphibians (including frogs, toads, and salamanders) often carry *Salmonella* bacteria in their intestines without any signs of clinical illness and can contaminate their surrounding environments through fecal shedding (*7*). Improper animal husbandry is a risk factor for human illness (*8*). If reptiles do not receive appropriate veterinary care, are fed an inappropriate diet, are housed in inappropriate environments (e.g., inadequate temperature, overcrowded, or environments in which they are unable to express natural behaviors or receive environmental cues), or are otherwise stressed during capture and transport, shedding of *Salmonella* bacteria can be exacerbated, increasing the risk for transmission to humans (*5*,*9*,*10*). Reptiles in captivity have indeed been noted to shed *Salmonella* at higher frequencies than their wild counterparts (*11*). Immunosuppression from husbandry stress, in snakes for example, has also been noted to change pet oral flora from predominantly gram-positive to aerobic gram-negative bacteria, increasing oral cavity colonization by *Salmonella* and potentially leading to more gastrointestinal tract shedding (*12*). Further, all adult amphibians and some reptiles (e.g., snakes) are obligate carnivores and require diets of live foods. Many examples of recommended feeding sources (e.g., crickets, worms, or rodents) (*13*) for carnivorous amphibians and reptiles are also natural reservoirs of *Salmonella*, further increasing the risk for infection in pet owners or those involved in animal husbandry who fail to take appropriate preventive measures (*5*).

Children <5 years of age, elderly persons, and those with impaired immunity are particularly susceptible to *Salmonella* because of their developing or weakened immune systems (*14*). Several outbreaks of reptile-associated salmonellosis have been reported in Canada and the United States, including recent outbreaks of *S. enterica* serovar Vitkin (bearded dragons, 2024), *S. enterica* serovar Lome (geckos, 2020–2024), *S. enterica* serovar Muenchen (geckos, 2020–2024), *S. enterica* serovar Cotham and *S. enterica* serovar Kisarawe (bearded dragons, 2012–2014), *S. enterica* serovar Typhimurium (snakes and feeder rodents, 2012–2014), and *S. enterica* serovar Enteritidis (snakes and feeder mice, 2012–2015); children accounted for a high proportion of cases in each instance (*15*–*20*). Similarly, numerous outbreaks of *Salmonella* Typhimurium linked to pet amphibians and turtles have been reported in the United States (*21*), including a large outbreak associated with dwarf clawed frogs (2008–2011) and a multistate outbreak associated with pet turtles (2008) (*22*,*23*). In both instances, children accounted for a high percentage of cases (*22*,*23*).

In Ontario, persons reported to have salmonellosis are interviewed by local public health investigators to identify a potentially causative source of infection. Where multiple possible exposures are reported, awareness of serotypes known to be associated with reptiles (or feeder prey) can aid in identifying the most likely exposure and inform follow-up actions. This study aimed to explore the proportion of locally acquired human salmonellosis case-patients in Ontario during 2015–2022 who reported reptile or amphibian contact before symptom onset, to assess the reptile species and *Salmonella* serotypes reported most often among those human case-patients, and to compare those observations to reptile and amphibian veterinary isolates reported during the same time period in Ontario, thereby establishing whether the reported reptile or amphibian exposure in human case-patients was a reasonable source of acquisition on the basis of correlating veterinary data.

## Methods

### Epidemiology

We extracted for analysis data for all human case-patients meeting the confirmed or probable case definition for salmonellosis in Ontario (24) and reported through the provincial electronic surveillance system by local public health units with an episode date of January 1, 2015–December 31, 2022. Episode date was captured per the following hierarchy depending on available information: symptom onset date, specimen collection date, laboratory test date, or date on which the case was reported to the local public health unit.

We extracted *Salmonella* serotype, demographic data, and exposure data for all human case-patients with a yes or no response regarding contact with reptiles or amphibians during the week before symptom onset. Sporadic cases had no epidemiologic link to a known common source. Outbreak-associated cases were linked by a shared spatiotemporal or exposure event or by PulseNet Canada whole-genome sequencing (WGS) with an initial threshold of 0–10 alleles by whole-genome multilocus sequence typing, and where >2 related isolates occurred within a 60-day period (*24*,*25*).

Per the standardized Ontario questionnaire for salmonellosis, persons who report travel during the exposure period do not necessarily need to be asked about other potential exposures, including reptile or amphibian contact; however, that information can be optionally collected (*26*). We thus excluded persons who reported travel during the exposure period from further analysis. Removing those cases was further justified because reptile and amphibian species can vary globally, and we anticipated interactions during travel to differ from those with pets or interactions through local petting zoos or wildlife exposure.

We compared demographic data for sporadic cases in which no travel was reported to assess differences in age or sex between those with or without reptile or amphibian contact. We performed descriptive analyses of case-patient demographics, *Salmonella* serotype, and reptile and amphibian types for a reduced dataset, which included sporadic case-patients who reported having contact with a reptile or amphibian in the week before symptom onset and who did not report travel. We used Pearson χ^2^ test to assess associations between case-patients reporting any or no reptile or amphibian contact and case-patient demographics or risk factors.

### Reptile and Amphibian Veterinary Isolates

We obtained Ontario reptile and amphibian veterinary isolate data reported to the Ontario Ministry of Agriculture, Food and Agribusiness (OMAFA) through mandatory reporting by veterinary diagnostic laboratories during 2015–2022 (*27*). Those data reflected all reptile and amphibian specimens submitted to Ontario veterinary diagnostic laboratories from private veterinarians as part of animal health examinations in which *Salmonella* was identified. Sequencing data from veterinary *Salmonella* isolates are not routinely reported to OMAFA; therefore, only serotype information was available for evaluation. We compared the serotypes detected with the human case data and further reviewed to assess the frequency of detection of specific serotypes by diet type and whether the animals were privately owned or from a zoological collection. We used the Pearson χ^2^ test to assess associations between reptile types and reptile diet. We conducted all analyses using Microsoft Excel (Microsoft) and SAS Enterprise Guide version 8.2 (SAS Institute Inc.) with a significance level of 5% (α = 0.05). This project did not require research ethics committee approval because the activities described herein were conducted in fulfillment of Public Health Ontario’s legislated mandate “to provide scientific and technical advice and support to the health care system and the Government of Ontario in order to protect and promote the health of Ontarians” and are therefore considered public health practice, not research (*28*).

## Results

### Epidemiology

A total of 18,452 human salmonellosis cases (all serotypes) were reported in Ontario during 2015–2022 (18,296 confirmed, 246 probable). Of those case-patients, 68.8% (12,585) had a yes or no response regarding reptile or amphibian contact in the week before symptom onset; the remainder reported contact as unknown or not asked. Most cases were sporadic (92.7%, 11,668/12,585); the remainder were linked to 1 of 97 unique outbreaks (ranging in size from 1–59 cases with a median of 6 cases). Of the outbreak-associated cases, 13 were linked by WGS to a known outbreak of *Salmonella* (I) 4,[5],12:i:- associated with snakes and feeder rodents (2022), 2 were linked by WGS to a known outbreak of *Salmonella* Vitkin associated with bearded dragons (2022), and the remainder were linked to nonreptile or feeder prey exposure.

Of all case-patients, 28.2% (3,551/12,585) reported travel outside of Ontario during the week before symptom onset and were excluded from subsequent analyses. Of the remaining sporadic case-patients, 6.3% (513/8,148) reported having contact with a reptile or amphibian in the week before symptom onset (from 4.3% [27/625] of cases in 2022 to 8.1% [99/1,225] of cases in 2015) (Table 1).

**Table 1:** Case-patient demographics for reported confirmed and probable sporadic locally acquired human cases of salmonellosis in Ontario, Canada, 2015–2022*

Characteristic	Reptile contact		Total cases	p value (χ^2^)
	Yes	No		
Sex†‡				0.37 (0.82)
M	234 (6.0)	3,637 (94.0)	3,871 (100.0)	
F	279 (6.5)	3,992 (93.5)	4,271 (100.0)	
All	513 (6.3)	7,629 (93.7)	8,142 (100.0)	
Age group, y‡§				<0.001 (46.1)
<1	46 (9.0)	278 (3.6)	324 (4.0)	
1–4	42 (8.2)	815 (10.7)	857 (10.5)	
5–9	62 (12.1)	553 (7.2)	615 (7.6)	
10–19	84 (16.4)	693 (9.1)	777 (9.5)	
20–29	109 (21.2)	939 (12.3)	1,048 (12.9)	
30–39	54 (10.5)	699 (9.2)	753 (9.2)	
40–49	52 (10.1)	696 (9.1)	748 (9.2)	
50–59	40 (7.8)	965 (12.6)	1,005 (12.3)	
60–69	13 (2.5)	853 (11.2)	866 (10.6)	
>70	11 (2.2)	1,142 (15.0)	1,153 (14.2)	
All	513 (100.0)	7,633 (100.0)	8,146 (100.0)	
Year				
2015	99 (8.1)	1,126 (91.9)	1,225	
2016	88 (6.2)	1,328 (93.8)	1,416	
2017	80 (6.4)	1,176 (93.6)	1,256	
2018	69 (5.3)	1,236 (94.7)	1,305	
2019	71 (6.4)	1,035 (93.6)	1,106	
2020	42 (6.5)	609 (93.5)	651	
2021	37 (6.6)	527 (93.4)	564	
2022	27 (4.3)	598 (95.7)	625	
All	513 (6.3)	7,635 (93.7)	8,148	

*Values are no. or no. (%) except as indicated. †Sex is reported in the provincial electronic surveillance system as male, female, transgender, or other. Analyses involving sex were restricted to data for case-patients with sex reported as male or female due to low counts for other variables. No case-patients with domestic acquisition of infection and reported reptile exposure reported sex other than male or female. ‡Sporadic cases only. §Of 513 case-patients with no travel history who reported reptile exposure.

Age was unknown in 2 sporadic cases with no reported reptile contact; we excluded those persons from analyses involving age. The age distribution of sporadic case-patients differed between those who reported having contact with a reptile or amphibian during the week before symptom onset and those who reported no contact (χ^2^ = 46.1; p<0.001) (Table 1). Of all sporadic case-patients who reported reptile or amphibian contact, 9.0% (46/513) were <1 year of age and 29.2% (150/513) were <10 years of age (Table 1). Comparatively, 3.6% of sporadic case-patients with no reported reptile or amphibian exposure were <1 year of age (278/7,635) and 21.6% were <10 years of age (1,646/7,635) (Table 1).

### Reptile and Amphibian Exposures

The most common reptiles reported by sporadic case-patients with local acquisition of infection were lizards (46.4%, 238/513) followed by snakes (26.7%, 137/513) and turtles or tortoises (19.3%, 99/513), whereas 10.7% (55/513) reported contact with amphibians (Table 1). Just under 12% of case-patients reported >1 reptile type (11.9%, 61/513). Corn snakes (*Pantherophis guttatus*) were the most frequently reported species of snake (44.1%, 15/34), followed by pythons (family Pythonidae) or ball pythons (*Python regius*) (32.4%, 11/34) and boas (family Boidae) (14.7%, 5/34). However, information regarding snake species was not collected for most case-patients reporting snake contact (75.2%, 103/137). Almost 12% of sporadic case-patients who reported reptile or amphibian contact did not have any details available regarding species (11.9%, 61/513), and 1 person reported contact with a crocodilian (0.2%, 1/513). Of those who reported contact with lizards, most reported contact with bearded dragons (*Pogona vitticeps*) (47.1%, 112/238), followed by other lizards (order Squamata) (29.4%, 70/238), geckos (order Gekkota) (26.1%, 62/238), chameleons (family Chamaeleonidae) (5.5%, 13/238), and iguanas (family Iguanidae) (3.8%, 9/238).

**Figure 1:**
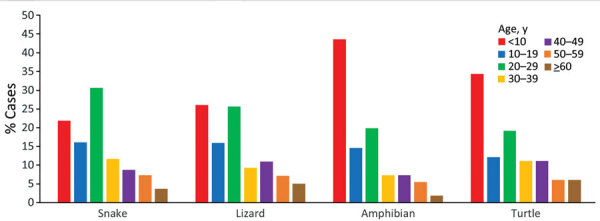
Age distribution of confirmed and probable sporadic locally acquired human salmonellosis case-patients who reported reptile or amphibian contact, by reptile type (n = 513), in study of reptile exposure among human cases and *Salmonella* serotypes isolated from reptiles, Ontario, Canada, 2015–2022. Reptile types reported by each case-patient were not mutually exclusive; thus, a case might be reported under >1 reptile type. The proportion of case-patients by age group is presented separately for each reptile type (i.e., percentages by age group for each reptile type sum to 100%).

Although we found no significant association between sex and whether a person reported contact with reptiles during the exposure period (χ^2^ = 0.82; p = 0.37), female patients were generally more likely than male patients to report contact with each reptile type and were significantly more likely to report contact with amphibians (Table 2). We noted a significant difference between age group and whether sporadic case-patients with local acquisition of infection reported reptile contact (χ^2^ = 46.1; p<0.001) (Table 1). We also found an apparent association between case-patient age and reptile type. Of those reporting contact with a snake, the largest percentage (30.7%, 42/137) were 20–29 years of age (Figure 1). Conversely, children <10 years of age made up the greatest percentages of those reporting contact with an amphibian (43.6%, 24/55) or a turtle (34.3%, 34/99) (Figure 1). Children <10 years of age (26.1%, 62/238) and adults 20–29 years of age (25.6%, 61/238) each made up a similar percentage of those reporting lizard contact (Figure 1).

**Table 2:** Case-patient reptile contact by sex for reported confirmed and probable sporadic locally acquired human cases of salmonellosis in Ontario, Canada, 2015–2022*

Reptile†	Case-patient sex‡		Total cases	p value (χ^2^)
	M	F		
Lizards	109 (45.8)	129 (54.2)	238	0.94 (0.006)
Snakes	58 (42.3)	79 (57.7)	137	0.22 (1.52)
Turtles	47 (47.5)	52 (52.5)	99	0.99 (0.0002)
Amphibians	17 (30.9)	38 (69.1)	55	0.01 (6.14)
Unspecified	32 (52.5	29 (47.5)	61	>0.99 (0.00)

*Values are no. or no. (%) except as indicated. †Of 513 case-patients with no travel history who reported reptile exposure. Sporadic cases only. Reported reptile types were not mutually exclusive. ‡Sex is reported in the provincial electronic surveillance system as male, female, transgender, or other. Analyses involving sex were restricted to data for case-patients with sex reported as male or female due to low counts for other variables. No case-patients with domestic acquisition of infection and reported reptile exposure reported sex other than male or female.

**Figure 2:**
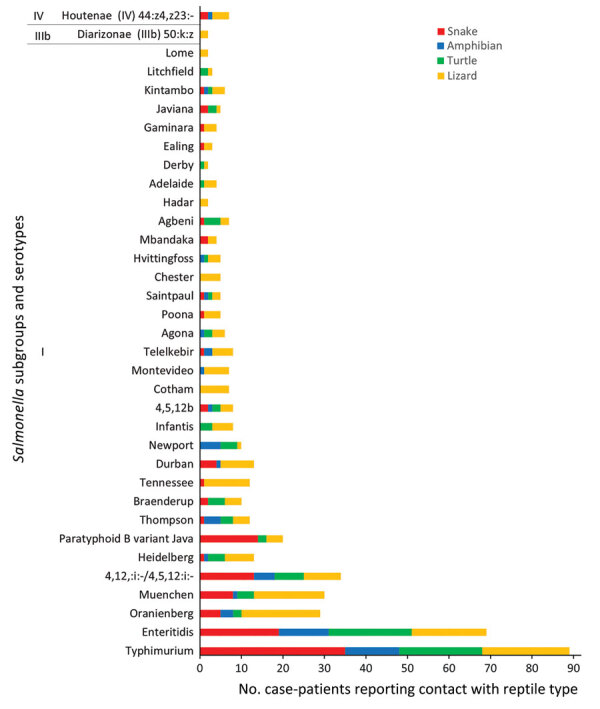
Major taxonomic grouping of reptile or amphibian contact reported by sporadic locally acquired salmonellosis cases by reported *Salmonella* serotype for human case-patients (n = 513) in study of reptile exposure among human salmonellosis cases and *Salmonella* serotypes isolated from reptiles, Ontario, Canada, 2015–2022. Reptile species were not mutually exclusive (i.e., some case-patients reported contact with >1 species); thus, reptile species counts may sum to more than the number of cases reported for each serotype over the study period.

A total of 94 unique *Salmonella* serotypes were reported, 47.9% (n = 45) of which were only reported once. Most human isolates (94.9%, n = 487) were *S*. *enterica* subsp. *enterica*. The most common serotypes identified among the sporadic human case-patients reporting reptile or amphibian contact were *Salmonella* Typhimurium (15.8%, 81/513), *Salmonella* Enteritidis (14.8%, 76/513), *Salmonella* Oranienburg (5.3%, 27/513), and *Salmonella* Muenchen (4.9%, 25/513), which are serotypes also often associated with feeder prey (Figure 2; Appendix Table) (*29*–*32*). Although some serotypes were associated with a range of reptile or amphibian exposures, some were more closely associated with a single type. For example, 87.5% (14/16) of *Salmonella* Paratyphi B biovar Java (also known as *Salmonella* Paratyphi B variant L [+] tartrate +) case-patients reported contact with a snake, whereas 100.0% (5/5) of *Salmonella* Chester, 81.8% (9/11) of *Salmonella* Tennessee, and 71.4% (5/7) of *Salmonella* Cotham case-patients reported contact with a bearded dragon and 80.0% (4/5) of *Salmonella* Agbeni case-patients reported contact with a turtle (Figure 2; Appendix Table). Most *S*. *enterica* subsp. *diarizonae* case-patients reported contact with lizards (57.1%, 8/14), as did most *S*. *enterica* subsp. *houtenae* case-patients (88.9%, 8/9).

### Reptile and Amphibian Veterinary Isolates

A total of 46 reptile submissions where *Salmonella* was detected were reported to OMAFA by veterinary diagnostic laboratories in Ontario during 2015–2022; 35 different serotypes were isolated. No amphibian submissions were reported to OMAFA during that period. Most animals were privately owned (80.4%, 37/46) versus zoological (19.6%, 9/46), and 89.1% (41/46) could be identified to the species level. Among privately owned reptiles, the most common species with a *Salmonella* isolate were bearded dragons (16.2%, 6/37), ball pythons (13.5%, 5/37), and corn snakes (8.1%, 3/37). The most common reptile species with *Salmonella* isolated from zoological collections was the Eastern Massasauga rattlesnake (*Sistrurus catenatus* [89%, 8/9]). More *Salmonella* isolates came from snake submissions (63.0%, 29/46) than from lizard submissions (34.8%, 16/46) or chelonian submissions (2.2%, 1/46) (χ^2^ = 16.73; p = 0.003).

**Figure 3:**
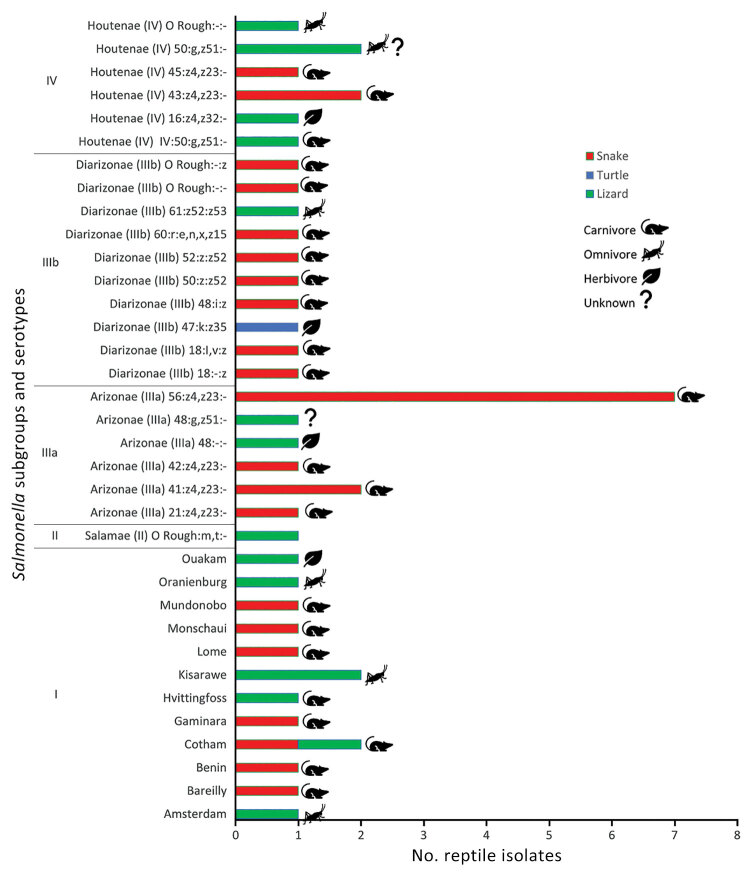
Major taxonomic grouping and diet of reptiles from which *Salmonella* was detected and reported to the Ontario Ministry of Agriculture, Food and Agribusiness by veterinary diagnostic laboratories in Ontario, by reported *Salmonella* serotype, in study of reptile exposure among human salmonellosis cases and *Salmonella* serotypes isolated from reptiles, Ontario, Canada, 2015–2022.

In this dataset, obligate carnivores were more likely to have *Salmonella* isolated (69.6%, 32/46) than were omnivores (15.2%, 7/46), herbivores (8.7%, 4/46), and species that could not be classified (6.3%, 3/46) (χ^2^ = 14.53; p<0.001) (Figure 3). For all reptile cases, the most common *Salmonella* serotype isolated was *Salmonella* IIIa:56:z4,z23:- (15.2%, n = 7/46), followed by *Salmonella* Cotham, *Salmonella* IIIa:41:z4,z23:-, *Salmonella* IV:43:z4,z23:-, *Salmonella* IV:50:g,z51:-, and *Salmonella* Kisarawe (4.3% for each, n = 2) (Figure 3). An additional 29 serotypes were detected only once (Figure 3).

## Discussion

As the popularity of reptiles kept as household pets has increased, a concurrent increase has occurred in the number of sporadic case-patients with *Salmonella* who report reptile contact before symptom onset, in addition to an increase in the number of outbreaks linked to reptiles and feeder rodents. Although only 2.1% of Ontario residents surveyed in 2014 reported having contact with a reptile in the previous week and 1.0% reported contact with an amphibian (*4*), 6.3% of sporadic *Salmonella* case-patients with no recent travel history reported contact with reptiles or amphibians in the week before symptom onset during the study period, reflecting a higher-than-expected frequency of reptile or amphibian contact among *Salmonella* case-patients compared with the background population. Several recent outbreaks of *Salmonella* have also been linked to reptiles (including snakes and lizards) and feeder rodents; children have been disproportionately affected (*15*,*19*).

Similar to reported trends in reptile ownership derived from online search data, lizards (particularly bearded dragons) and snakes were commonly reported by Ontario case-patients with reptile exposures (*33*). The finding that *Salmonella* case-patients reporting reptile or amphibian contact were generally younger than those with no reported reptile contact likely reflects young children’s increased susceptibility and increased risk for exposure to bacteria because of a combination of developing immune systems, limited hand hygiene, tendency to mouth their hands and objects, and lack of supervision during reptile contact (*14*). Observed associations between age and reptile type may reflect differences in pet ownership; however, low counts for some age groups resulted in insufficient study power to reliably test for a statistical difference between age groups. Recognizing the risk for *Salmonella* infection associated with pet turtle ownership (particularly for turtles with a shell <4 cm long, which children might be more likely to handle and place in their mouths), the US Food and Drug Administration banned the sale of small turtles to the public (i.e., as pets) in 1975; however, no such national ban exists in Canada, and any ownership restrictions are decided at a municipal level (*34*,*35*). Despite the US ban on turtles sold as pets, national outbreaks linked to small turtles sold illegally continue to occur, reflecting enforcement challenges associated with such bans (*21*).

Although the most common reptile species with detection of *Salmonella* reported to OMAFA were similar to the most common reptiles reported by human salmonellosis case-patients, the most frequent *Salmonella* serotypes isolated were dissimilar. In the reptile veterinary cases, greater diversity was observed in the *Salmonella* serotypes isolated. In our study, this finding might be explained by the veterinary patient population included; identified serotypes might reflect those more likely to be isolated from reptiles experiencing stress or illness (and hence, reptiles undergoing veterinary examination) than isolates from apparently healthy animals sampled during human health investigation. *Salmonella* serotype richness and prevalence have been observed to increase in reptiles after shipment; those changes are typically attributed to exposure to stress, among other factors (*36*). Most *Salmonella* serotypes identified from reptile veterinary isolates belonged to *S. enterica* subsp. *enterica*, *arizonae*, and *diarizonae*, and within each subspecies many serotypes are known to be reptile-associated (*2*,*37*).

A carnivorous diet (*10*) was associated with greater risk for *Salmonella* isolation from reptile veterinary cases in this dataset. Several outbreaks of human salmonellosis associated with contact with snakes and feeder rodents have been documented, which might also underlie an increased risk for veterinary salmonellosis cases in reptiles fed contaminated prey (*18*,*38*). Feeding live prey contaminated with *Salmonella*, combined with poor husbandry, poor welfare, or other concurrent physiologic stressors such as debilitation through inanition and dehydration in wild-caught reptiles, might lead to increased *Salmonella* shedding and subsequent contamination of the cage enclosure with *Salmonella* (*3*,*5*). Many omnivorous and herbivorous reptiles also consume their own fecal matter as part of their normal diet or use fecal marking as part of territoriality and home range marking, which could amplify infection.

Previous research (*39*) noted that 48% of pet reptiles sampled from private households and pet shops were found to carry *Salmonella*, and a literature review (*3*) found that captive reptiles had higher rates of *Salmonella* carriage than did wild reptiles. Similar to other research, we found that *Salmonella* was isolated more frequently in snakes than in lizards or chelonians (*3*,*11*,*37*,*39*). Snakes are highly prone to *Salmonella* infections because of rodent-based diets and captive husbandry practices, which could explain their association with human disease (*12*). Pet owners and others in contact with reptiles should adopt precautionary measures while handling reptiles, feeder prey, and their enclosures, assuming that *Salmonella* could be present even if the reptiles appear healthy (*40*). Routine culture of *Salmonella* could be difficult in healthy reptiles, and treatment to reduce carriage is not possible; therefore, routine pet testing might not be advisable unless persons at risk for severe illness are frequently exposed or unless reptile handling is part of a commercial business (*12*). In the event of an outbreak of human salmonellosis in which reptiles or amphibians are suspected to be the source of exposure, collection of environmental swab samples from the animal’s enclosure might successfully identify the same serotype. In fact, isolating *Salmonella* from environmental specimens was found to be more reliable than isolating *Salmonella* from animal fecal specimens, possibly because of intermittent fecal shedding (*15*,*19*,*40*). Furthermore, as part of human outbreak management, pet owners should be encouraged to seek appropriate veterinary care, including husbandry analysis, to resolve immunocompromised health status, given pet owners might have a poor understanding of optimal health and welfare of their pets (*41*).

Although we identified associations between reptile contact and subsequent human illness, we did not adjust analyses for other potentially causative exposures reported during the exposure period (i.e., consumption of specific food items or other animal exposures). Thus, reptile contact should not necessarily be inferred to be the causative exposure responsible for human illness. Data used for analyses were subject to case-patient recall and individual investigator interview and data entry practices; thus, some outbreak-associated cases might have been misreported as sporadic. Ideally, public health investigators should ensure that all *Salmonella* case-patients are asked about reptile and amphibian contact, including reptile type. If a human case or outbreak of *Salmonella* infection involving a species other than *S*. *enterica* is identified, potential reptile- or amphibian-associated exposure should be considered. Similarly, if reptile or amphibian exposure is reported during a *Salmonella* case or outbreak investigation and a reptile-associated serotype is identified, further testing should be considered to confirm the source of illness and inform case education and outbreak prevention.

The lack of amphibian animal salmonellosis cases in this study and overall far fewer isolations of *Salmonella* from reptiles compared with isolations in humans with exposure to reptiles suggest that *Salmonella* carriage might not always lead to illness in reptiles and amphibians, signs of illness might not always be recognized, veterinary care might not always be available, or the cause of death might not always be investigated (*42*). In developing educational resources for pet owners, consideration should be given to providing information to pet owners on the risks associated with both reptile ownership and handling of feeder prey such as rodents. Reminding pet owners that reptiles kept as pets should receive regular veterinary care, both to ensure the health of reptiles and to provide opportunities for ongoing pet owner education, is also crucial.

Appendix Additional information about reptile exposure in human salmonellosis cases and *Salmonella* serotypes isolated from reptiles, Ontario, Canada, 2015–2022
