# Supplementary material for: Reptile Exposure in Human Salmonellosis Cases and Salmonella Serotypes Isolated from Reptiles, Ontario, Canada, 2015–2022
Source: Emerg Infect Dis. 2025 Oct;31(10):1912–21. doi: 10.3201/eid3110.241803 (PMC12483020; doi:10.3201/eid3110.241803)
Supplement: Appendix — Additional information about reptile exposure in human salmonellosis cases and Salmonella serotypes isolated from reptiles, Ontario, Canada, 2015–2022 [file 24-1803-Techapp-s1.pdf]

Article DOI: https://doi.org/10.3201/eid3110.241803

EID cannot ensure accessibility for supplementary materials supplied by authors. Readers who have difficulty accessing supplementary content should contact the authors for assistance.

Reptile Exposure in Human Salmonellosis Cases and *Salmonella* Serotypes Isolated from Reptiles, Ontario, Canada, 2015–2022

Appendix

Appendix Table. Total number of human case-patients reporting contact with each reptile type, and total number of reptile isolates from which *Salmonella* was isolated, by reported *Salmonella* serotype (2015–2022)*

Subgroup	Serotype	Snake†	Snake contact	Lizard†	Lizard contact	Turtle†	Turtle contact	Amphibian†	Amphibian contact	Total reptile isolates	Total human isolates
I	4,12,:i:- / 4,5,12:i:-	0	13	0	9	0	7	0	5	0	29
	4,5,12b	0	2	0	3	0	2	0	1	0	7
	Adelaide	0	0	0	3	0	1	0	0	0	3
	Agbeni	0	1	0	2	0	4	0	0	0	5
	Agona	0	0	0	3	0	2	0	1	0	5
	Amsterdam	0	0	1	0	0	0	0	0	1	0
	Bareilly	1	0	0	1	0	0	0	0	1	1
	Benin	1	0	0	0	0	0	0	0	1	0
	Braenderup	0	2	0	4	0	4	0	0	0	12
	Chester	0	0	0	5	0	0	0	0	0	5
	Cotham	1	0	1	7	0	0	0	0	2	7
	Derby	0	0	0	1	0	1	0	0	0	3
	Durban	0	4	0	8	0	0	0	1	0	10
	Ealing	0	1	0	2	0	0	0	0	0	3
	Enteritidis	0	19	0	18	0	20	0	12	0	76
	Gaminara	1	1	0	3	0	0	0	0	1	3
	Hadar	0	0	0	2	0	0	0	0	0	4
	Heidelberg	0	1	0	7	0	4	0	1	0	19
	Hvittingfoss	0	0	1	3	0	1	0	1	1	5
	Infantis	0	0	0	5	0	3	0	0	0	8
	Javiana	0	2	0	1	0	2	0	0	0	3
	Kintambo	0	1	0	3	0	1	0	1	0	3
	Kisarawe	0	0	2	2	0	0	0	0	2	2

Subgroup	Serotype	Snake†	Snake contact	Lizard†	Lizard contact	Turtle†	Turtle contact	Amphibian†	Amphibian contact	Total reptile isolates	Total human isolates
	Litchfield	0	0	0	1	0	2	0	0	0	3
	Lome	1	0	0	2	0	0	0	0	1	3
	Mbandaka	0	2	0	2	0	0	0	0	0	5
	Monschaui	1	0	0	0	0	0	0	0	1	0
	Montevideo	0	0	0	6	0	0	0	1	0	7
	Muenchen	0	8	0	17	0	4	0	1	0	25
	Mundonobo	1	0	0	0	0	0	0	0	1	0
	Newport	0	0	0	1	0	4	0	5	0	10
	Oranienburg	0	5	1	19	0	2	0	3	1	27
	Ouakam	0	0	1	0	0	0	0	0	1	0
	Paratyphi B var. Java	0	14	0	4	0	2	0	0	0	16
	Poona	0	1	0	4	0	0	0	0	0	6
	Saintpaul	0	1	0	2	0	1	0	1	0	6
	Telelkebir	0	1	0	5	0	0	0	2	0	7
	Tennessee	0	1	0	11	0	0	0	0	0	11
	Thompson	0	1	0	4	0	3	0	4	0	14
	Typhimurium	0	35	0	21	0	20	0	13	0	81
II	Salamae (II) O Rough:m,t:-	0	0	1	0	0	0	0	0	1	0
IIIa	Arizonae (IIIa) 56:z4,z23:-	7	0	0	0	0	0	0	0	7	0
	Arizonae (IIIa) 41:z4,z23:-	2	1	0	0	0	1	0	0	2	1
	Arizonae (IIIa) 48:-:-	0	0	1	0	0	0	0	0	1	0
	Arizonae (IIIa) 48:g,z51:-	0	0	1	0	0	0	0	0	1	0
	Arizonae (IIIa) 21:z4,z23:-	1	0	0	0	0	0	0	0	1	0
	Arizonae (IIIa) 42:z4,z23:-	1	0	0	0	0	0	0	0	1	0
IIIb	Diarizonae (IIIb) 61:z52:z53	0	0	1	1	0	0	0	0	1	1
	Diarizonae (IIIb) 18:l,v:z	1	0	0	0	0	0	0	0	1	0
	Diarizonae (IIIb) 18:-:z	1	0	0	0	0	0	0	0	1	0
	Diarizonae (IIIb) 48:i:z	1	0	0	0	0	0	0	0	1	0
	Diarizonae (IIIb) 50:k:z	0	0	0	2	0	0	0	0	0	3
	Diarizonae (IIIb) 50:z:z52	1	0	0	0	0	0	0	0	1	0
	Diarizonae (IIIb) 52:z:z52	1	0	0	0	0	0	0	0	1	0
	Diarizonae (IIIb) 60:r:e,n,x,z15	1	0	0	0	0	0	0	0	1	0
	Diarizonae (IIIb) O Rough:-:-	1	1	0	0	0	0	0	0	1	1
	Diarizonae (IIIb) O Rough:-:z	1	0	0	0	0	0	0	0	1	0
	Diarizonae (IIIb) 47:k:z35	0	0	0	0	1	0	0	0	1	0
IV	Houtenae (IV) 50:g,z51:-	0	0	1	2	0	0	0	0	1	2
	Houtenae (IV) 43:z4,z23:-	2	0	0	0	0	0	0	0	2	0
	Houtenae (IV) 16:z4,z32:-	0	0	1	0	0	0	0	0	1	0
	Houtenae (IV) O Rough:-:-	0	0	1	0	0	0	0	0	1	0
	Houtenae (IV) 44:z4,z23:-	0	2	0	4	0	0	0	1	0	4
	Houtenae (IV) IV:50:g,z51:-	0	0	2	0	0	0	0	0	2	2
	Houtenae (IV) 45:z4,z23:-	1	0	0	0	0	0	0	0	1	0
	Total	29	136	16	204	1	216	0	55	46	448

*Note that reptile species were not mutually exclusive (i.e., some cases reported contact with more than one species), thus the number of human case-patients reporting contact with each reptile type may sum to more than the number of human cases reported for each serotype over the study period. Only those *Salmonella* serotypes identified in one or more reptile submissions, and/or reported for 3 or more human cases are shown here.

‡Reptile types from which each *Salmonella* serotype was identified.
